# From mutation to mechanism: deciphering the molecular function of genetic variants linked to human ageing

**DOI:** 10.1093/bfgp/elab005

**Published:** 2021-03-05

**Authors:** Maarouf Baghdadi, Helena M Hinterding, Linda Partridge, Joris Deelen

**Keywords:** genetic variants, lifespan, healthspan, longevity, functional characterisation, model organisms

## Abstract

Many of the leading causes of death in humans, such as cardiovascular disease, type 2 diabetes and Alzheimer’s disease are influenced by biological mechanisms that become dysregulated with increasing age. Hence, by targeting these ageing-related mechanisms, we may be able to improve health in old age. Ageing is partly heritable and genetic studies have been moderately successful in identifying genetic variants associated with ageing-related phenotypes (lifespan, healthspan and longevity). To decipher the mechanisms by which the identified variants influence ageing, studies that focus on their functional validation are vital. In this perspective, we describe the steps that could be taken in the process of functional validation: (1) *in silico* characterisation using bioinformatic tools; (2) *in vitro* characterisation using cell lines or organoids; and (3) *in vivo* characterisation studies using model organisms. For the *in vivo* characterisation, it is important to focus on translational phenotypes that are indicative of both healthspan and lifespan, such as the frailty index, to inform subsequent intervention studies. The depth of functional validation of a genetic variant depends on its location in the genome and conservation in model organisms. Moreover, some variants may prove to be hard to characterise due to context-dependent effects related to the experimental environment or genetic background. Future efforts to functionally characterise the (newly) identified genetic variants should shed light on the mechanisms underlying ageing and will help in the design of targeted interventions to improve health in old age.

## Introduction

Life expectancy has been steadily rising in the world, partly due to treatment of the elderly but mostly due to the reduction of early life mortality and treatment of communicable disease [1]. The increasing population of elderly individuals will bring a concomitant increase in multimorbidity [2, 3]. Stagnating birth rates and a growing percentage of pensioners are posing a serious challenge to our economies and will do so to an even greater extent in the future. Data from the European Union highlight how age-associated multimorbidity leads to a rise in individual healthcare expenditure on older people, up to 18% per capita gross domestic product [4]. Furthermore, healthcare costs differ between sexes, mainly due to differences in multimorbidity and lifespan, with women using a third more resources than men and the majority of human healthcare expenditure taking place in middle to old age [5]. The use of an overwhelming amount of capital to develop and test therapies targeting age-associated diseases, such as cancer, with only marginal benefits in quality-adjusted life years (average 2 months) [6], raises the question whether resources would be better spent targeting the underlying biological mechanisms dysregulated with age rather than disease-related endpoints [7]. Moreover, by focusing on the compression of morbidity, societies can benefit from the tremendous social and economic opportunities that come with an active and vibrant older population [8].

The idea of reducing multimorbidity by targeting ageing comes from the fact that exceptionally long-lived individuals and their family members often present a compression of morbidity or a longer lifespan free of disease [9, 10]. Nevertheless, they suffer from the same causes of death at old age (i.e. they do not seem to be immune to disorders but rather have a later onset of disease) [9]. There is evidence suggesting that the factors contributing to these benefits are partly heritable, given that longevity [i.e. survival to an exceptional old age (e.g. top 10% of their respective birth cohort)] can be transmitted as a quantitative genetic trait [11]. On the other hand, the evidence for a genetic component of lifespan (i.e. number of years lived), an alternative phenotype used to study ageing, is more compelling. In twin studies for lifespan, the heritability has been estimated to be around 25% [12]. However, large genealogical studies for lifespan offer a more modest view of heritability (i.e. below 12%) [13, 14], potentially due to the diverse population studied (geographically diverse), inclusion of early mortality and accounting for the non-additive genetic component. Taken together, there is enough support for studying ageing-related phenotypes using genetic approaches. Ultimately, the aim of genetic studies on ageing is to identify genes that can elucidate mechanisms of healthy physiological ageing, which can subsequently be targeted using lifestyle and/or pharmacological interventions to reduce (multi)morbidity.

**Figure 1:**
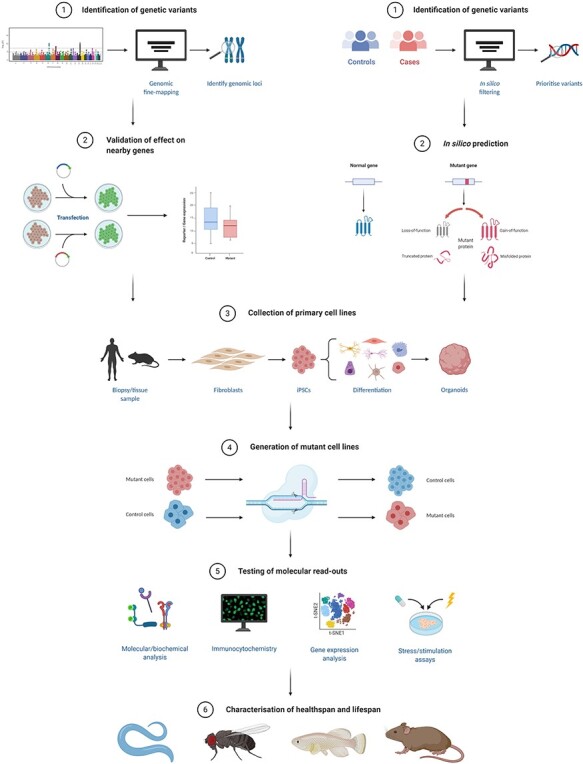
Pipeline for functional characterisation of genetic variants linked to human ageing.

In this perspective, we will give a short overview of the current state of research on the genetics of ageing and provide suggestions, as well as future directions, for functional characterisation of variants identified in genetic studies (Figure 1).

## Genetics of ageing

To decipher the genetic component of ageing, genetic studies have investigated a variety of phenotypes linked to ageing, such as lifespan, healthspan (i.e. number of years lived before onset of an age-related disease) and longevity. The most commonly used approach to study these phenotypes has been through genome-wide association studies (GWAS). These GWAS have identified several loci associated with ageing-related phenotypes when analysed separately [15], and even more when combined together [16]. However, it is important to provide functional evidence for the implicated loci to validate their credibility and understand their mode of action.

Due to the relatively small sample size of GWAS of ageing-related phenotypes, these studies are only able to detect variants that are relatively common in the population [minor allele frequency (MAF) >1%]. However, given the polygenic pleiotropic nature of the genetic landscape underlying ageing [17], the field has begun to move towards the identification of rare genetic variants (MAF <1%) involved in ageing using whole-genome/exome sequencing approaches. A similar approach is also applied to other complex diseases and traits [18, 19]. To maximise the power of these studies, they focus on sequencing of the most extreme cases in the population (i.e. exceptionally long-lived individuals and/or members of long-lived families). The number of long-lived individuals included in sequencing studies to date is still quite small [20–22], which limits their statistical power to detect single genetic variants associated with longevity [23]. Therefore, these studies mostly focus on genetic variation in candidate genes guided by results from model organism–based studies. Thus far, these efforts have provided rare functional variants in two genes: *FOXO3* [24] and *IGF1R* [25]. There are several large sequencing efforts ongoing, which should be able to provide additional rare genetic variants relevant for ageing. However, because of the aforementioned limited statistical power, it is critical to provide functional evidence for rare genetic variants identified through sequencing studies.

## Functional characterisation

### In silico

Often, a locus identified through GWAS contains multiple genetic variants associated with the studied trait. To determine which of these variants are the most likely functional variants within a locus, several steps can be taken (Figure 1), as reviewed in detail elsewhere [26]. Many of the genetic variants identified through GWAS are found in intronic or intergenic regions, leading to difficulties in assigning a clear biological function to the variant. The variants must be carefully evaluated, as non-coding variants can have significant effects on nearby or distant genes via transcriptional, post-transcriptional or post-translational mechanisms. The many available bioinformatic tools and online resources could provide hints by annotation of transcription factor binding sites, chromatin structure and regulatory elements (enhancers, promoters and repressors). For example, the effect of a genetic variant on expression of genes in different tissues can be assessed through GTEx (https://gtexportal.org/home/). Given that most of the effects on gene expression are tissue specific, the *in silico* characterisation can also provide valuable input for subsequent *in vitro* and *in vivo* experiments.

In contrast, a sequencing study will define the genetic variant of interest from the start, and these variants are rare and often only observed in a heterozygous state. However, due to the increase in resolution, many more variants are identified, making it difficult to narrow down the list into a practical number for functional validation. Therefore, most studies make use of predetermined criteria to ensure unbiased filtering of genetic variants (Figure 1). Filtering steps that are usually applied are: (1) incorporating knowledge from previous studies on model organisms (i.e. selection of candidate genes/pathways) using online resources (https://genomics.senescence.info/genes/) and literature review; (2) simulating the effect of variants on a protein domain structure or function by using online tools such as CADD [27] or more advanced molecular modelling [28–30]; (3) establishing the frequency of the variant (i.e. the variant should be absent or have a very low frequency in the general population) using publicly available reference databases, such as GenomAD (https://gnomad.broadinstitute.org/), DiscovEHR (http://www.discovehrshare.com/) and MGRB (https://sgc.garvan.org.au).

### In vitro

Cell lines are a common tool used to explore functional effects *in vitro*. As many genetic variants identified through GWAS are found in non-coding regions, most *in vitro* studies first focus on measurements that can be used to determine their effect on enhancer/promoter activity, with luciferase and transcription factor binding assays, or transcription of surrounding genes, using qPCR or more advanced techniques (Figure 1) [31–34]. An example of successful *in vitro* follow-up of a genetic variant coming from genetic studies of ageing is provided by Grossi and colleagues, who have shown that a variant in *FOXO3*, residing within an enhancer region, creates a binding site for HSF1 that results in increased transcriptional activity of *FOXO3* and its target genes in response to oxidative stress [35]. Due to the lack of genetic conservation of non-coding regions in model organisms, many of the genetic variants coming from GWAS cannot be moved forward to an *in vivo* model. Therefore, the *in vitro* characterisation concludes the functional validation of these variants.

While primary cells from long-lived individuals would serve as the gold standard for *in vitro* characterisation (Figure 1), such cells are often difficult to obtain and culture. One example of using primary cells is by reprogramming them into induced pluripotent stem cells (iPSCs). This allows for the study of the effect of a genetic variant on a cellular phenotype, such as stress resistance, in the context of an individual’s genetic background. An advantage of investigating cells from long-lived individuals is that epistatic effects are also assessed, although it is harder to pinpoint the functional effect to a specific genetic variant. Moreover, the differentiation potential of iPSCs into various cell and tissue types offers the advantage of studying tissue-specific functional effects [36]. One should, however, take into account that reprogramming leads to modification of a cell’s epigenetic landscape, which could influence ageing-related molecular read-outs such as gene expression [37]. 3D *in vitro* organoid culture is another method to mimic cellular organization, intercellular communication and crucial extracellular matrix interaction, with a closer approximation to the physiological microenvironment of a given tissue than traditional cell culture methods [38]. To overcome the practical limitations of primary cell lines, researchers often turn to cancer cell lines and genetic engineering tools to study a variant’s effect in an adjustable environment. This is done by comparing cells with the introduced genetic variant to its control counterpart to assess gain- or loss-of-function effects (Figure 1). This approach has, for example, been applied by Tazearslan and colleagues to determine the functional effect of two genetic variants in *IGF1R* identified by sequencing a cohort of long-lived individuals [25, 39]. However, the identified heterozygous variants were only assessed in the homozygous state. Therefore, the potential role of compensatory effects in the presence of the wild-type protein still needs to be addressed.

The *in vitro* functional characterisation and validation of genetic variants have advanced immensely since the practical application of the CRISPR/Cas9 gene editing technology. Initially discovered in bacteria as a defence mechanism against viruses [40], scientists have learned to exploit and adapt this technique in order to create targeted knockouts and site-specific point mutations in a wide array of cell types. Essentially, only two components are needed: a complementary guide RNA unique to a locus of interest and a Cas9 enzyme that cuts the DNA sequence. These steer the cell’s repair mechanism towards endogenous non-homologous end joining or a cleaner version of homology-directed repair by providing a DNA repair template [41]. Recent advances have also modified the Cas9 protein to be more specific in targeting [42], thereby minimizing the potential of off-target modifications obscuring a variant’s phenotype. Despite the advantages of cell models for functional characterisation of genetic variants, they also pose certain limitations. For example, genetic background effects and inherent genomic instability in cell lines could hide the functional effects under investigation. Furthermore, many of the widely used cell lines are derived from immortalized cancer cells and possess karyotypic abnormalities, such as polyploidy, that could affect both the genetic engineering methods as well as downstream functional analyses [43].

Assessing the effect of the genetic variant on its host protein and direct downstream targets, for example by looking at stability, functional sites (e.g. phosphorylation), expression and transcription, using, for example, immunohistochemistry, qPCR or pull-down assays, can serve as a guide for the subsequent in-depth functional characterisation (Figure 1). As a next step, the obtained or created cell lines can be functionally characterised using ageing-related cellular and molecular read-outs based on the hallmarks of ageing [44]. In Table 1, we have provided an overview of assays that can be used to study the effects of a variant on the different hallmarks *in vitro*. Moreover, stress assays (e.g. H_2_O_2_, UV and heat) can be used to determine hallmark-overarching effects [45].

**Table 1:** Overview of assays that can be used to study the hallmarks of ageing *in vitro*

Hallmark of ageing	*In vitro* assays
Genomic instability	● Base excision repair capacity [46, 47] ● Measuring DNA lesions [48]
Cellular senescence	● Beta-galactosidase [49], p53 and p16 stainings to assess mitotic arrest [50]
Mitochondrial dysfunction	● Basal mitochondrial respiration [51] or substrate-uncoupler-inhibitor titration protocols [52] ● qPCR to asses mtDNA copy number [53] ● Assessing the integrated stress response after doxycycline treatment [54, 55]
Loss of proteostasis	● LysoTracker [56] ● LC3 (immunohistochemical) staining to assess autophagic flux [57] ● Citrate synthase activity assay for chaperone activity [58]
Epigenetic alterations	● DNA methylation (arrays/bisulfite sequencing) [59]
Stem cell exhaustion	● Stemness markers (immunofluorescence) or proliferation assays such as HALO-96 PREP [60]
Telomere attrition	● Terminal restriction fragment (TRF) [61] ● Quantitative fluorescence in situ hybridization (Q-FISH) [61] ● qPCR [61]
Deregulated nutrient-sensing and altered intercellular communication	● Assessment of IIS/mTOR activity after nutrient deprivation (i.e. serum or amino acid starvation) or stimulation (e.g. with insulin, IGF-1 or EGF) by immunohistochemistry/immunoblotting [62, 63]

IIS, insulin/insulin-like growth factor-1 signalling; mTOR, mammalian target of rapamycin; IGF-1, insulin-like growth factor-1; EGF, epidermal growth factor.

### In vivo

*In vitro* experiments provide invaluable mechanistic information but lack critical insight on how different tissues respond to a genetic intervention or how the effect of a genetic variant changes with age. A unique opportunity of genetic studies in model organisms, other than conducting lifespan analyses, is the possibility of performing longitudinal healthspan assays that can shed light on biological mechanisms of resilience used by healthy ageing individuals to combat the hallmarks of ageing [64] (Table 2). Performing measurements at multiple time points across the life of an animal allows the assessment of progressive decline in an outcome measure as, for example, shown for motor ability and sleep [65, 66]. It is important to include time points with enough temporal resolution in an ageing study to prevent misinterpretation of the results, especially if future therapeutics will be designed to intervene in the age-related trajectory that is indicative of the function of a gene or pathway [67]. The end point for validating a genetic variant associated with ageing is vague and open to discussion. However, it is clear that this variant must at least have an effect, either alone or in combination with other variants, on healthspan. Currently, we do not have any proxies for overall healthspan *in vitro*. Given that ageing is a time-dependent cumulative process where disease risk increases, this property must ideally be assessed in the process of functional validation.

**Table 2:** Overview of advantages, limitations and studied health outcomes of different model organisms used for the *in vivo* characterisation of genetic variants linked to ageing

Model organism	Lifespan (days)		Advantages	Limitations	Health outcome
	Median	Max			
**Nematode worm** [70] *Caenorhabditis elegans*	~ 15	~ 27	● Large brood size ● Short lifespan and generation time ● Several distinct tissues ● Transparent body ● Comprehensive genetic toolbox ● Easy and inexpensive culture and handling ● Easy storage and genetic line maintenance	● Does not replicate human organ systems ● No conservation in the genome ● Post-mitotic adult tissue (except germline) ● Invertebrate (no skeletal system) ● Males only have one sex chromosome ● No clear circadian rhythm	● Muscle loss [85] ● Tissue decline [86, 87] ● Nucleolar size [88] ● Body bends or thrashing [89] ● Pharyngeal pumping rate [89]
**Fruit fly** [90] *Drosophila melanogaster*	~ 80	~ 100	● Large brood size ● Short lifespan and generation time ● Several distinct tissues ● Comprehensive genetic toolbox ● Easy and inexpensive culture and handling ● Sex-specific studies possible ● Diurnal	● Does not replicate all human organ systems ● Limited conservation with humans in the genome ● Post-mitotic adult tissue (except intestine) ● Invertebrate (no skeletal system) ● Cannot be recovered alive from freezing so time-consuming storage and line maintenance	● Neuromuscular (climbing) [91] ● Circadian rhythm dysregulation (sleep) [66] ● Gut integrity, female specific [92] ● Fecundity, female specific [93]
**African turquoise killifish** [94] *Nothobranchius furzeri*	~ 121	~ 243	● Large brood size ● Genome engineering possible [95] ● Vertebrate ● Longitudinal assessment of individual animals possible ● Easy storage and maintenance of lines ● Sex-specific studies possible ● Diurnal	● No standardized healthspan parameters yet ● Limited conservation in non-protein coding genome ● Sufficient tissue homology (closed circulatory system/innate and adaptive immunity) ● Requires special facility for maintenance and filtration ● Lack of developed genetic toolbox ● Requires ethical approval	● Kyphosis (back arching) [77] ● Muscle loss [77] ● Wound repair [77] ● Colour loss [77] ● Fecundity [96]
**Mouse** [97] *Mus musculus*	~ 730	~ 1460	● High genome conservation ● Comprehensive genetic toolbox ● Vertebrate ● Longitudinal assessment of individual animals possible ● Easy storage and maintenance of lines ● Sex-specific studies possible ● Diurnal ● Placental viviparity	● Relatively long lifespan ● Limited conservation in non-protein coding genome ● Good tissue homology ● Expensive upkeep ● Requires special facility for maintenance ● Nocturnal—tests performed during daytime are not ideal ● Requires ethical approval	● Cognition (NOR) [98] ● Neuromuscular function [83] ● Motor coordination [83] ● Metabolic status (energy balance/body composition) [83] ● Metabolic health (GTT/ITT) [83] ● Cardiac function [83] ● Gate speed [99] ● Non-invasive frailty index [100]

NOR, novel object recognition; GTT, glucose tolerance test; ITT, insulin tolerance test.

After enough evidence has been obtained from the *in vitro* experiments, the natural next step is to introduce the genetic variant into a model organism in which the ageing process can be characterised and an investigation can be launched into its role on lifespan or healthspan modulation (Figure 1). The model organism of choice is variant dependent, as genetic conservation, tissue homology and practical reasons may favour some organisms over others. Below we will highlight some of the most commonly used model organisms in genetic studies of ageing. We have focused on healthspan outcomes that exhibit functional decline with age and that can be assessed non-invasively.

#### Nematode worms

The nematode worm (*Caenorhabditis elegans*) has been the de facto model organism for the study of the genetics of ageing, since the field was founded by paradigm shifting work in which a single mutation resulted in doubling of the worm’s lifespan [68]. The short lifespan of worms coupled with the ability to perform genetic screens for traits has led to many insights into genetic mechanisms that regulate lifespan, with many pathways implicated in higher-order organisms [69]. The balance between the presence of multiple distinct tissues and simplicity (lack of redundancy) of genetic pathways has led to an enticing experimental model system. Moreover, the transparent nature of worms allows non-invasive imaging to track the effect of reporter-tagged genetic modifications throughout their lifespan. Additionally, scientists have leveraged the worm’s transparency to assess different healthspan parameters, such as age-associated tissue decline, nucleolar size and body bends (Table 2) [70]. Moreover, worms provide a powerful method for performing gene knockdown in the whole organism as well as in a spatially restricted manner by feeding them RNAi-generating bacteria for assessment of gene function [71]. Finally, with the advent of machine learning and accompanying technological advancements, scientists have developed automated methods for performing lifespan assays allowing for high-throughput genetic studies.

#### Fruit flies

The fruit fly (*Drosophila melanogaster*) is another great tool for ageing research due to its relatively short lifespan, practical husbandry and advanced genetic tools. These genetic tools, for example, allow elegant and precise spatiotemporal control of genetic perturbations. This allows studies to address questions about how genetic modifications affect tissue-specific functional decline or tissue–tissue interactions during the ageing process. Tissues in fruit flies are more homologous to humans than those in worms but still lack homology in metabolic organs such as liver and pancreas. However, flies possess a brain with a diversity in cell types similar to mammals. Moreover, the fruit fly is an invertebrate with a robust circadian rhythm that declines with age, allowing investigation of the association of circadian dysregulation and ageing [66]. Unlike worms, fruit flies possess heteromorphic sex chromosomes and the ability to determine the sex of individual cells in a cell-autonomous manner, allowing the study of important mechanisms of sexual dimorphism without confounding effects of circulating sex hormones [72]. Large numbers of animals can be assessed in lifespan assays to investigate the effect of a mutation on the mortality rate, providing greater insight into the gene function than just mean and maximum lifespan. Moreover, the assessment of mortality rate is important in determining if a genetic intervention leads to a change in age-specific mortality or age-independent mortality, potentially indicative of whether any increase in lifespan is attributable to slowed ageing or a general improvement in health [73]. The rich history of studies in fruit flies has brought forward many well-developed healthspan assays for which ageing trajectories have already been described, such as climbing and sleep (Table 2) [65, 66]. The combination of all these advantages and more (Table 2) makes fruit flies another valuable tool to study the biological mechanisms of ageing [74].

#### African turquoise killifish

The African turquoise killifish (*Nothobranchius furzeri*) is a relatively new model organism that is gaining significant popularity in the ageing field. These killifish are an enticing middle ground between the short lifespans of invertebrate models and the developed organ system of vertebrates, such as an adaptive immune system. Previous invertebrate models possess short maximal lifespans (Table 2), but typical vertebrate models have a maximum lifespan of over 4 years, prohibiting repeated results, iteration of experiments and reducing feasibility of studies trying to verify novel ageing genes. There has been a great effort to develop the genetic and genomic toolkit of killifish, opening the door to the genetic modification of this unique vertebrate model organism [75]. The killifish is a relatively new model organism, and so the healthspan measures are still under development and currently limited to visual macroscopic inspection of the animal (Table 2). However, there are already studies reporting the development of cognitive and locomotor assays that are modulated by environmental ageing interventions [76]. Overall, the killifish is an interesting model organism to incorporate into functional genetic studies of ageing given its unique properties [77].

#### Mice

Mice (*Mus musculus*) are a great model organism for studying human pathology and longevity as 99% of mouse genes have a sequence match in the human genome [78]. However, mice have a dramatically shorter lifespan, there are outstanding genetic tools available and there is potential to perform invasive assays [67]. Additionally, mice are social animals with a rich behavioural repertoire, allowing scientists to assess complex social interactions, which are known to influence both mortality and morbidity throughout life [79, 80]. Because of these unique advantages, mice have provided great insight into the biological mechanisms of ageing [81]. Unfortunately, measuring healthspan is challenging, as multiple organ systems need to be assessed across the lifespan of the organism, especially as ageing is mediated by pleiotropic genes. Typically, studies focus on one or two organ systems and study them in great detail. However, in the field of gerontology, it is important that overall health is assessed and that this is done in both sexes, if possible, as differences between sexes have been observed in the natural ageing process and in response to interventions (see Table 2 for examples) [82].

Recently, a great effort has been made by major labs in Europe and the United States to develop a standard operating procedure for longitudinal healthspan assessment in mice, targeting a variety of organ systems, to increase robustness, reproducibility and utility [83]. The introduction of the National Institute of Aging’s multi-institutional Interventions Testing Program (ITP), with the aim of investigating lifespan and healthspan extending interventions, is a clear effort of cooperation and aspiration towards reproducible investigation [84].

## Translational follow-up studies

Once the functional characterisation of a genetic variant is complete, the next step would be to try to mimic the health-promoting effects of the variant using targeted lifestyle and/or pharmacological interventions. While model organisms are invaluable for the mechanistic understanding of proposed interventions, their genetic and environmental characteristics could reduce the relevance of the experimental results to humans. For example, the major cause of death for mice is cancer [101], while for humans, it is ischaemic heart disease, followed by stroke [102]. As a result, treatment with agents that target age-related diseases in mice, such as rapamycin, which reduces cancer growth, may prove to be less effective in humans. An approach to address whether the interventions that improve healthspan in model organisms are likely to be relevant to humans is the use of species that are more genetically related to humans, such as non-human primates, or that share the human environment, such as companion dogs. These organisms display many of the age-related phenotypes and diseases observed in humans and could therefore provide insight into the translatability of interventions that show promising results in model organisms [103, 104]. Initial short-term studies using rapamycin have demonstrated healthspan-promoting effects in the common marmoset (*Callithrix jacchus*) [105, 106] and in companion dogs (*Canis lupus familiaris*) [107], with long-term lifespan and healthspan studies already planned or in progress. A potential disadvantage of using non-human primates is their relatively long lifespan. However, recently introduced model species such as the grey mouse lemur (*Microcebus murinus*) and common marmoset are relatively short lived (average lifespan of 7–10 years), which allows longitudinal studies within a reasonable time frame.

The final step in the process would be to test the effectiveness of the identified health-promoting interventions in humans. However, before reaching this stage, analysis of data collected from carriers of the functional genetic variants may already provide insights into specific metabolic profiles associated with healthy ageing. The depth of these kinds of analyses, often referred to as phenome-wide association studies [108], depends a lot on the frequency of the variant under investigation and, hence, such analyses are often only feasible for variants identified through GWAS. It is important that studies in humans include individuals from different ancestries to make sure that the identified mechanisms are broadly shared and the targeted lifestyle and pharmacological interventions could be applied to the population as a whole.

## Conclusion

Recent advances in the field have resulted in the identification of several genetic variants associated with healthy ageing. Moreover, the availability of affordable sequencing is pushing the field into the direction of identification of rare variants (in candidate genes/pathways). However, given that genetic studies are not able to provide information about causality, it is important to provide functional evidence for such variants using *in silico*, *in vitro* and, ideally, *in vivo* tools. The point at which a variant shows enough evidence to be considered causally involved in healthy ageing is still under debate, especially if *in vivo* characterisation is not possible due to the absence of conservation of the variant. We have tried to provide an overview of outcomes that could be used to determine the functional effect of a variant, but some effects may be context specific [i.e. only visible in a certain genetic background (due to epistasis), sex or environmental state]. With the continuous development of gene editing tools, we will soon also be able to test multiple variants at the same time [109], which will at least allow the study of additive and epistatic effects. Moreover, the inclusion of genetically heterogeneous mice in *in vivo* studies, as is currently done in the ITP [101], will allow the study of genetic variants in a diverse but reproducible genetic background. Given the polygenic nature of ageing, we do not expect to find one shared mechanism among genetic variants ‘explaining it all’, but rather a variety of mechanisms each influenced to a mild extent by one or a few genetic variants. Once health-promoting mechanisms have been identified, future studies should focus on the development of lifestyle and pharmacological interventions targeting these mechanisms. To make sure that findings from model organisms can be translated to humans, it is important to harmonize phenotypes and focus on biomarkers that can be assessed non-invasively. This will allow for quick iteration of interventions in humans without having to wait for terminal outcomes like mortality and (multi)morbidity. Biomarkers of the ageing process that translate well across humans and mice, such as frailty [100], have also proven to respond to health-promoting pharmacological interventions [110]. The most straightforward tissue to study is blood given that this is easy to collect in humans and has the unique property that it is in contact with all the organs, including the brain [111], and can thereby provide a good overview of an individual’s health status in a non-invasive manner. Blood can thus be used as a bridge between model organism– and human-based studies to investigate the effects of an intervention on health-promoting mechanisms. Examples of blood-based biomarkers of ageing that can be included in studies of model organisms are those coming from studies of the human epigenome, proteome and metabolome [112–114].

**Key Points**

- By studying the genetic components of ageing, we may be able to identify mechanisms that could be targeted by lifestyle and pharmacological interventions to improve healthy ageing in the general population.
- Genetic studies of ageing-related phenotypes have identified multiple genetic variants associated with ageing.
- Functional characterisation of genetic variants is required to prove causality and reveal mechanisms.
- The depth and breadth of functional characterisation (i.e. *in silico*, *in vitro* and/or *in vivo*) depend on the conservation of the genetic variant in model organisms and context-specific effects (e.g. epistasis or environmental state).
- *In vivo* studies in model organisms should focus on phenotypes related to both lifespan and healthspan with a focus on translational outcomes.

## Notes

**Maarouf Baghdadi** is a PhD student at the Max Planck Institute for Biology of Ageing. His PhD project is focused on understanding the sex-specific effect of genetic interventions related to insulin signalling in mammalian models.

**Helena M. Hinterding** is a PhD student at the Max Planck Institute for Biology of Ageing. Her PhD project is focused on exploring the role of genetic variation in the MAPK/ERK signalling pathway in human longevity.

**Linda Partridge** is the founding director of the Max Planck Institute for Biology of Ageing. Her research is focused on evolutionarily conserved dietary, genetic and pharmacological interventions promoting healthy ageing.

**Joris Deelen** is a research group leader at the Max Planck Institute for Biology of Ageing. His research is focused on the genetics and biomarkers of healthy ageing in humans.
